# Neuroplasticity of Acupuncture for Stroke: An Evidence-Based Review of MRI

**DOI:** 10.1155/2021/2662585

**Published:** 2021-08-19

**Authors:** Jinhuan Zhang, Chunjian Lu, Xiaoxiong Wu, Dehui Nie, Haibo Yu

**Affiliations:** ^1^The Fourth Clinical Medical College of Guangzhou University of Chinese Medicine, Shenzhen 518033, China; ^2^Shenzhen Traditional Chinese Medicine Hospital, Shenzhen 518033, China

## Abstract

Acupuncture is widely recognized as a potentially effective treatment for stroke rehabilitation. Researchers in this area are actively investigating its therapeutic mechanisms. Magnetic resonance imaging (MRI), as a noninvasive, high anatomical resolution technique, has been employed to investigate neuroplasticity on acupuncture in stroke patients from a system level. However, there is no review on the mechanism of acupuncture treatment for stroke based on MRI. Therefore, we aim to summarize the current evidence about this aspect and provide useful information for future research. After searching PubMed, Web of Science, and Embase databases, 24 human and five animal studies were identified. This review focuses on the evidence on the possible mechanisms underlying mechanisms of acupuncture therapy in treating stroke by regulating brain plasticity. We found that acupuncture reorganizes not only motor-related network, including primary motor cortex (M1), premotor cortex, supplementary motor area (SMA), frontoparietal network (LFPN and RFPN), and sensorimotor network (SMN), as well as default mode network (aDMN and pDMN), but also language-related brain areas including inferior frontal gyrus frontal, temporal, parietal, and occipital lobes, as well as cognition-related brain regions. In addition, acupuncture therapy can modulate the function and structural plasticity of post-stroke, which may be linked to the mechanism effect of acupuncture.

## 1. Introduction

Stroke is a common disease that affects one in four people during their lifetime [1], globally, and it continues to be a leading cause of death and long-term disability worldwide, imposing a significant financial burden on healthcare systems and families [2, 3]. Although stroke incidence and prevalence have declined worldwide, however, a recent national epidemiological survey [4, 5] indicated that China has an estimated 11 million prevalent cases of stroke, 2.4 million new cases of stroke, and 1.1 million stroke-related deaths. Hemiparesis and aphasia are two of the prominent impairments caused by a stroke that affect activities of daily living activities and quality of life [6–8]. More than 80% of poststroke patients experience upper or lower limb hemiplegia, severely disturbing their daily activities [9]. Some studies have found that almost 20%-40% of all stroke survivors have chronic aphasic symptoms [10, 11]. It is well known that returning to work and social activities is the key priority for stroke survivors. Therefore, it is critical to understand stroke pathogenesis and explore its appropriate treatment.

Previous studies [12, 13] have demonstrated that poststroke patients have structural and connectivity changes in their brains. Luckily, the brain's plasticity, a broad term for the proof the human brain to adapt to environmental pressure, experiences, and challenges including brain damage [14, 15], enables stroke rehabilitation. Although many patients experience some degree of spontaneous recovery, that is, a time-determined amount of improvement in physical function and activity [16], it is often incomplete and the recovery rates of neurological function vary. Therefore, external stimulus interventions are still needed. However, despite extensive research efforts on multiple treatment modalities, no single rehabilitation intervention has been demonstrated to be definitively beneficial for recovery [17]. Even the most commonly used repetitive transcranial magnetic stimulation (rTMS) and transcranial direct current stimulation (tDCS) were not recommended for routine stroke treatment in two Cochrane reviews [18, 19]. Due to a lack of effective therapy, researchers considered alternative approaches that improve stroke recovery. As a relatively inexpensive and safe treatment, acupuncture has been widely employed to improve motor, sensation, and some neurological functions of stroke for thousands of years. Furthermore, several clinical [20–22] research and systematic reviews [17, 23, 24] revealed that acupuncture, as a promising intervention, could improve motor and language function and daily living activities. Numerous studies [25–27] have suggested that plasticity and reorganization contribute to the recovery.

However, the current understanding of neuroplasticity after stroke is primarily based on invasive methods, such as histology and immunohistochemistry, which do not allow for dynamic assessment of functional recovery and tissue remodeling [28]. In contrast, magnetic resonance imaging (MRI) can noninvasively monitor dynamic change after stroke and in vivo. Structural magnetic resonance imaging (sMRI) technique can provide a high anatomical resolution [29], whereas functional magnetic resonance imaging (fMRI) can reveal real-time brain activity by indirect measurement of regional blood flow [30]. Combined with sMRI and fMRI, the central nervous effect of acupuncture for stroke could be fully elucidated from an anatomical and functional perspective. Moreover, emerging clinical studies have demonstrated that acupuncture could reorganize motor-related networks and increase functional connectivity between premotor cortex (PM)/adjacent supplementary motor area (SMA) and supramarginal gyrus (SMG) [31–33]. In addition, acupuncture therapy has various properties, such as the choice of acupoints, whether deqi or not, which may be the influencing factors of acupuncture on the plasticity of stroke patients.

Nevertheless, the underlying neuroplasticity mechanisms on acupuncture for stroke have received little attention to date. Therefore, the review will mainly focus on the evidence to elucidate the possible mechanisms of acupuncture therapy in treating stroke through regulating brain plasticity based on MRI to better select and stratify patients for future appropriate treatment strategies that promote poststroke recovery. We firstly describe research characteristics of acupuncture for stroke based on MRI. Then, we discuss the neuroplasticity mechanism of acupuncture and its properties on stroke. Furthermore, we also review the limitations and prospects to be explored in the future.

## 2. Materials and Methods

We conducted a literature search for MRI studies on acupuncture for stroke published in PubMed, Web of Science, and Embase from inception to April 9, 2021. Database searches were conducted using the following keywords: (acupuncture OR electroacupuncture OR moxibustion) AND (stroke OR cerebral ischemia OR ischemic cerebrovascular disease OR hemiparesis or hemiplegia OR post-stroke) AND (MRI OR magnetic resonance imaging OR functional MRI and structural MRI OR BOLD OR ReHo OR ALFF OR fALFF OR white matter OR voxel-based analysis OR VBM OR voxel-based morphometry OR Freesurfer OR surface-based morphometry OR cortical thickness OR surface area OR cortical volume OR gray matter volume OR gray matter density OR DTI). Studies were eligible if they met the following inclusion criteria: (1) randomized controlled trials (RCTs) and nonrandomized studies (i.e., observational studies, case-control studies, and cohort studies); (2) patients met established diagnostic criteria of stroke; (3) subjects in the study at least underwent MRI of the brain on one occasion: under the acupuncture state or before and after acupuncture treatment. We excluded studies that met the following criteria: (1) protocol, case reports, or case series. (2) Other interventions that do not belong to traditional acupuncture, such as transcutaneous electrical nerve stimulation and transcutaneous vagus nerve stimulation. (3) Comorbid severe mental illness or neurological illness.

All identified studies were imported into EndNote; duplicate studies were removed first, and then after scanning titles and abstracts and reading the full text, eligible studies were decided whether they should be included in the review.

**Figure 1:**
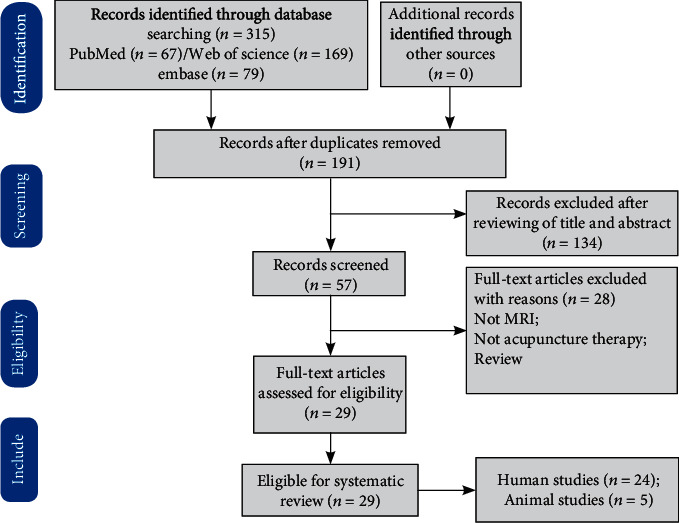
PRISMA flow diagram. Note: PRISMA: preferred reporting items for systematic reviews and meta-analyses.

Two authors extracted the following data: publishing year, author, number of participants, type of ischemic stroke, intervention/control groups, needling details, types of acupuncture, acupuncture points, data analysis, and experimental design. Any inconsistencies were discussed and resolved with the third author until an agreement is reached. Twenty-four human studies and five animal studies were finally included (Figure 1).

In this review, 24 stroke patient studies were included that use MRI to investigate the mechanism of acupuncture for stroke. The results indicated that acupuncture could modulate brain plasticity in motor-related and language-related networks of stroke patients. For acupuncture modality, 20 studies applied manual acupuncture (MA), and two studies used electroacupuncture (EA). Stroke types were found to be related to ischemic stroke. The publication years ranged from 2006 to 2020, indicating that research on this aspect has gradually become a research hotspot over the last 15 years. The sample size of the study ranged from 7 to 43 (mean 24). The study design mainly has several kinds: before vs. after acupuncture, acupuncture vs. sham acupuncture (SA), acupuncture vs. waiting group, patients vs. healthy controls (HC), and acupuncture plus drugs/conventional therapy vs. drugs/conventional therapy. In addition, except for five studies [34–38] with resting-state (RS) and long-term effects, all other studies investigated task-stating and instant effects. The detailed characters of included studies are listed in Table 1. In addition, five animal experiments also were included. The years of publication ranged from 2011 to 2021, and the study design mainly includes middle cerebral artery occlusion (MCAO) vs. MCAO plus EA group vs. sham operation group and EA vs. non-EA group. The detailed characters of the included studies are listed in Table 2.

## 3. Modulation of Brain Plasticity in Stroke

Stroke alters the landscape of the brain and impairs the function of various systems and structures [59]. One of the most striking features of the brain is its ability to adapt to external and internal stimuli. Indeed, several decades ago, Hebb [60] puts forward a theoretical framework that described the phenomenon of brain adaptation to the environment based on experience and development. The theories of neuroplasticity showed that thinking and learning change both the brain's physical structure and functional organization. Basic mechanisms that are involved in plasticity include neurogenesis, programmed cell death, and activity-dependent synaptic plasticity [61]. As the research progresses, neural plasticity is a general term that refers to functional and structural changes that occur in the brain during development, interaction with the environment, aging, learning, and in response to trauma [62, 63]. Adult brain plasticity following stroke is due to numerous diffuse and redundant connections in the central nervous system and the ability to form new structural and functional circuits through remappings between related cortical regions [64]. MRI has the advantage of providing repeated whole-brain measurements, making it ideal for longitudinal studies of network-level brain plasticity [62].

Brain plasticity occurs at many levels from molecules to cortical reorganization [27]. Advances in MRI technology have allowed system-level monitoring of brain structure and function in vivo. Functional plasticity can be detected through changes in the strength of functional interactions between brain regions, whereas structural changes can be identified in vivo indirectly and nonspecifically via sMRI measures [62].

Pathologically, damage to regions of the motor-related cerebral cortex, such as primary motor area (M1), premotor area (PMA), supplementary motor area (SMA), somatosensory area (S1), prefrontal cortex (PFC), and posterior parietal cortex (PPC) [65], results in hemiplegia. In contrast, damage to regions of the left perisylvian network, including inferior frontal gyrus (IFG), middle frontal gyrus (MFG), angular gyrus (AG), supramarginal gyrus (SMG), superior temporal gyrus (STG), middle temporal gyrus (MTG), inferior temporal gyrus (ITG), and supplementary motor area (SMA), leads to aphasia. Fortunately, a large body of research evidence indicates that the brain recovers rapidly and reorganizes its structure and function following a stroke. In other words, specific linguistic impairments caused by stroke showed substantial recovery in the first few months following a stroke [66], and hemispheric interactions have complex effects on the recovery of brain function after stroke [67, 68].

As research on poststroke recovery increases, one meta-analysis [69] of motor-related neural activity after stroke included 36 studies and demonstrated that consistently activated regions include contralesional primary motor cortex (M1), bilateral ventral premotor cortex, and supplementary motor area (SMA) compared with healthy controls (HC). Interestingly, this is consistent with another meta-analysis [9], which investigated the modulation of interhemispheric activation balance (IHAB) in stroke patients with motor recovery and demonstrated that IHAB is upregulated in sensorimotor cortex(SMC) and premotor cortex (PMC), but not significantly changed in SMA and cerebellum (CB). In addition, several studies also investigated the underlying mechanism of language processing in aphasia and found that early stroke patients showed significantly decreased functional connectivity (FC) in the language network [70]. Rs-fMRI studies also revealed a significant correlation between disrupted functional connectivity and the severity of poststroke language impairment [71].

## 4. Brain Plasticity in Stroke with Acupuncture

Brain plasticity provides a critical theoretical basis for central nervous system therapy [72]. In this review, 24 human and five experimental studies investigated the neuroplasticity mechanism of acupuncture in treating poststroke motor impairment, motor aphasia, and cognitive impairment from different analytical methods, study, and experimental designs based on MRI. We summarized the findings based on analytic methods and different rehabilitation aspects.

### 4.1. Stroke Patients' Studies

FC provides one method based on a system-level approach to quantify the functional integration of various brain regions by correlating brain activity to detect neural interactions between regions, which are quite compelling [73]. Moreover, FC analyses can provide experience-dependent plasticity at the macro level of large-scale functional networks, which are foundational to remediation interventions that maximize function recovery [74]. FC of the three studies used M1 as the region of seed interest, and the results revealed that acupuncture increased FC between left primary motor cortex (M1) and right M1, premotor cortex, supplementary motor area (SMA), thalamus, and cerebellum.

The Granger causality analysis is used to analyze the flow of information between time series, which has been widely used in the field of neuroscience [75]. A study [32] used the multivariate Granger causal analysis method and found that acupuncture induced a concentrated and bidirectional enhancement in effective connectivity between cerebellum and primary sensorimotor cortex in stroke patients. In addition, acupuncture probably integrated the effective connectivity internetwork by modulating multiple networks and transferring information between left frontoparietal network (LFPN) and sensorimotor network (SMN) by default mode network (aDMN and pDMN) as the relay station [51].

Graph theoretical analysis provides an uncomplicated but powerful mathematical framework to describe topological properties of brain networks, such as modularity, efficiency, and hubs [76, 77]. A study [54] using this method found that acupuncture could modulate the disrupted patterns of the whole-brain network following stroke, elucidating the possible mechanisms underlying the functional reorganization of poststroke brain networks following acupuncture intervention from a large-scale perspective.

Regional homogeneity (ReHo) is used to evaluate signal synchronization by calculating the time-series similarity in BOLD signals within local brain regions [78]. Acupuncture was found to increase ReHo values in the right precentral gyrus and superior frontal gyrus while decreasing them in the right superior parietal lobule, left fusiform gyrus, and left supplementary motor area.

Apart from that, voxel-based-morphometry (VBM) and diffusion tensor imaging (DTI) are popular structural MRI technique to investigate regional differences in brain volume and microstructural integrity [79, 80]. According to some studies [36, 37], acupuncture could lead to pronounced structural reorganization in frontal areas and network of DMN areas and increase fractional anisotropy (FA) values.

In addition, the plasticity function of acupuncture on stroke is manifested not only in motor function but also in language. Acupuncture was found to activate language-related brain areas, including frontal, temporal, parietal, and occipital lobes, as well as insula, precuneus, and other wide range of brain function areas.

In order to determine the efficacy of acupuncture, the design of comparison mainly has three kinds: stroke vs. HC, VA vs. SA, and VA plus drug vs. drug. Results demonstrated that VA compared with SA, VA plus drug compared with drug, and acupuncture in patients compared with that in HC all have the characteristics of remodeling the brain structure and function of stroke patients.

### 4.2. Animal Studies

Each of the five animal studies investigated the mechanism of acupuncture for stroke; among them, four studies focus on poststroke motor impairments, while the fourth examines poststroke cognitive impairment.

Wen et al. [58] investigated the effect of EA for middle cerebral artery occlusion induced cognitive deficit (MICD) group and found that brain infarction volume was reduced and ALFF was decreased in auditory cortex, cingulate gyrus, lateral nucleus group of dorsal thalamus, and hippocampus after 14 days of treatment.

One study [55] using DWI (diffusion-weighted imaging) indicated that the mechanism by which EA can treat acute stroke may be by reducing cerebral edema. While Wu et al. [56] found that acupuncture improved motor function, brain microscopy using DTI technique.

A recent study [81] showed that EA could decrease the infarct volumes of MCAO rats, improve mNSS scores, and enhance FC between the left motor cortex and left cerebellum posterior lobe, right motor cortex, left striatum, and bilateral sensory cortex.

ReHo also was used to investigate the regional neural activity alterations of stroke, and the results showed that EA could increase ReHo in auditory and motor cortex, lateral nucleus group of dorsal thalamus, hippocampus, and others [57].

Briefly, the above studies demonstrated that acupuncture promotes stroke-related neural plasticity from structural and functional aspects. The rehabilitation mechanism of acupuncture on stroke patients may be linked to the remodeling of motor and cognitive brain regions such as motor cortex, bilateral striatum, and sensory cortex hippocampus.

### 4.3. Factors Associated with the Brain Plasticity of Acupuncture

In this review, the influencing factors of acupuncture on stroke plasticity based on MRI include the type of SA, deqi, different acupoints, and different pathological states.

Compared to SA, four studies [34, 37, 41, 45, 46] demonstrated that VA produces a greater maximum activation change in the motor-related area, improves blood flow to ischemic areas, and promotes stroke recovery. However, one study [46] discovered significant impact variations between VA and SA at TE5, but little difference between verum acupoint and nonacupoint, implying that different SA types also have distinct brain responses.

Eight studies [32, 33, 39, 40, 42, 44, 47, 49, 54] have compared the differences in brain plasticity between stroke patients and HC and found that the modulation effect of acupuncture on stroke patients was more specific and more obvious than that of HC in brain regions associated with disease.

In terms of the choice of acupoints, the most commonly used acupuncture points are mainly in the limbs, and the most frequently used acupoints are GB34 and SJ5.

One study [48] investigated the central mechanism of deqi of acupuncture in the treatment of ischemic stroke and found that compared with the non-deqi group, the deqi group produced marked activation of the right anterior lobe of the cerebellum and right limbic lobe.

## 5. Discussion

In this review, we included 24 human studies and 5 animal studies and found that that acupuncture reorganizes not only motor-related network, M1, SMA, sensorimotor network (SMN), FA, aDMN, and pDMN but also language-related brain areas include inferior frontal gyrus frontal, temporal, parietal and occipital lobes as well as cognition-related brain regions. In addition, the plasticity of acupuncture is influenced by deqi, acupoints, and physiological state.

### 5.1. Brain Plasticity of Acupuncture

Stroke causes not only local structural changes in the injured brain regions but also damage to neuronal networks, impairing sensation, movement, or cognition [64, 82]. Under physiological conditions, both hemispheres inhibit each other, and after a stroke, this balance of interaction/inhibition may be upset due to damage to one side of the brain. Moreover, recent studies [83, 84] have demonstrated that interhemispheric imbalance is closely related to the motor function of the affected hand in chronic stroke patients. Thus, several studies [31, 42, 43, 46] have indicated that acupuncture could inhibit contralesional brain activity while activating the ipsilesional motor cortex. Huang et al. [43] indicated that acupuncture results in lateralization in unilateral stroke patients. This lateralization may represent an enhancement of the compensatory process through acupuncture that redistributes function to the intact cortex, especially the unaffected hemisphere. In addition, studies [44, 52] also showed that acupuncture could stimulate bilateral regions, modulate whole-brain network, and enhance functional connectivity. This indicated that acupuncture could not only specifically regulate the bilateral dynamic balance of the brain but also modulate the whole brain network and functional connections as a whole.

The pathogenesis of stroke is complex, the time since stroke, lesion size, location, and other biological factors (such as age and sex) all contribute to the differences between individuals. Therefore, in clinical practice, individualized treatment is based on TCM syndrome differentiation theory, the theory of constitution, and characteristics of patient, season, and locality. However, among the included studies, the reason the current study did not apply individualized therapy is that it requires big data to explore the impact of patients, doctors, and acupuncture on efficacy. In the future, the use of artificial intelligence coupled with continuous monitoring should enable greater individualization and improve outcomes. Importantly, although different acupoints were used across studies, they all could remodel brain areas associated with stroke lesions. For instance, several studies [31, 33, 51, 52] have demonstrated that acupuncture could enhance FC of between bilateral M1s, between the cerebellum and primary sensorimotor cortex, which indicated that acupuncture has not only specific but also common effects on the disease.

Additionally, as described above, although acupuncture therapy can modulate the function and structural plasticity of poststroke in this review. Indeed, structural plasticity has been explored in only one study for the following reasons: on the one hand, the study did not detect structural changes in stroke patients; on the other hand, there was a change in structural plasticity following acupuncture, but the small changes were difficult to identify due to lacking of subdivision of brain regions.

### 5.2. Factors Associated with the Brain Plasticity of Acupuncture

This review found that different SA types also have distinct brain responses. The overlapping dermatomes between nonacupoints and verum acupoints may explain this phenomenon, as the segmental structure of the body and its interconnected reflex system offers neurophysiological effects [85]. Therefore, the selection of proper SA is critical in determining the efficacy of acupuncture.

When it comes to acupoint selection, the most frequently used acupoints are GB34 and SJ5. GB34 is located on the lateral aspect of the posterior knee, which is the most often used acupoints to generally improve symptoms in motor impairment patients. GB34 belongs to the sea point of gallbladder meridian of foot Shaoyang. SJ5 is found in the dorsal wrist lines on two inches between the ulna and radius. SJ5, belonging to Sanjiao Meridian of Hand Shaoyang, is one of “Ba-mai Jiao-hui point.” The two acupoints have been widely used to alleviate symptoms in motor impairment patients. Although the two acupoints were located in the upper and lower extremities, the activated brain regions included bilateral brain, such as somatosensory cortex and primary sensorimotor. This indicated that various acupoints treat the same disease in a convergent manner.

For decades, it was believed that the deqi of acupuncture is associated with its clinical efficacy. Using fMRI techniques in acupuncture research, several studies [86, 87] have found that acupuncture with deqi can stimulate significant brain activity compared to acupuncture without deqi. In this review, although the deqi group exhibited significant activation of the right anterior lobe of the cerebellum and right limbic lobe (BA30), larger sample sizes are still required for further validation.

Regarding different states and based on TCM theory, this review found that acupuncture showed specific modulations of a motor-related network in stroke patients relative to HC. This phenomenon is in line with the TCM theory that acupuncture can regulate the disorder of the body in dual-direction regulation. This indicated that acupuncture in patients mainly regulate the brain regions associated with the disease, while acupuncture in HC mainly activated brain areas directly associated with the main treatment effects.

In the aspect of different states, based on TCM theory, acupuncture can regulate the disorder of the body in dual-direction regulation. Acupuncture exhibits distinct regulatory effects on the body under physiological and pathological conditions. This indicated that acupuncture showed specific modulations of a motor-related network in stroke patients relative to HC.

In summary, the brain plasticity of acupuncture on stroke is influenced by many factors, such as deqi, acupoints, pathological state, and SA type. As a result, it is important to continue exploring the most effective strategies for treating stroke with acupuncture in the future.

### 5.3. Prospects for Brain Plasticity of Acupuncture

Numerous clinical and experimental researches revealed that the brain was plastic and could be remodeled by the environment and experience [88, 89]. Currently, the National Institutes of Health in the United States has recently adopted acupuncture as a treatment for poststroke rehabilitation, demonstrating that acupuncture is widely accepted for such therapy [90]. From a systematic level, this study found that the effective mechanism is that acupuncture can reorganize the brain structure and functional connections in stroke patients.

Indeed, acupuncture's function to reshape the brain is not limited to stroke, as a recent review [91] of brain plasticity in animals revealed that acupuncture could modulate the plasticity of various central nervous systems, such as depression, neuropathic pain, Alzheimer's disease, and cerebral vascular disorders. More research in humans still requires further verification.

In addition, this review stated that acupuncture's plasticity on stroke was affected by several factors, such as deqi, acupoints, pathological state, and type of sham needle. Exploring the impact of these influencing factors on the efficacy and constructing a pathway connecting “acupoint-brain” is also critical for future individualized therapy. Moreover, predicting the efficacy of individual patients receiving acupuncture treatment for stroke to achieve precision treatment impact is a problem that requires future research. The recent integration of machine learning (ML) and neuroimaging techniques provides a promising approach to understanding how acupuncture facilitates neuroplasticity at the individual level. This approach enables us to investigate not only the effect of acupuncture influencing factors on brain plasticity prediction but also the impact of specific brain plasticity on acupuncture efficacy prediction.

Interestingly, a recent review [92] examined the neuroplasticity of acupuncture using machine learning and neuroimaging techniques and found that brain functional plasticity is affected by different acupoints and acupuncture manipulations and that specific structural and functional neuroplasticity characteristics at baseline could accurately predict the improvement of symptoms after acupuncture treatment. This review summarizes two commonly used methods for predicting the efficacy of acupuncture. One method is to adopt the classification algorithms to predict patients' responses to acupuncture treatment. The other method is to construct the regression models to predict the continuous improvement in symptoms after acupuncture treatment. Currently, this is the mainly used medication for pain and functional dyspepsia. Since research in this field remains in its infancy and faces many challenges, many efforts remain to be done in the future.

Apart from that, although several reviews [93, 94] have been published on acupuncture on stroke in animals at the molecular level, there have been a few animal studies on MRI-based acupuncture in stroke treatment. One possible reason is that the MRI mechanism is easy to manipulate in humans, unlike studies at the cellular and molecular levels. The other reason is that experimental stroke models do not fit perfectly into clinical situations, influencing the extrapolation of results. However, animal research also exhibits several advantages, including low costs, small variations, controllable factors, and high reproducibility. As a result, additional experimental studies may be required to elucidate the mechanism of influencing acupuncture factors on stroke.

### 5.4. Limitations

Although this review comprehensively summarizes the evidence from extensive MRI-based literature on acupuncture for stroke, several limitations remain. First, the main criticism is of this review is that study design, experimental design, and analytic methods may influence the extrapolation of conclusions, which also makes it difficult to do a meta-analysis. More relatively consistent designs and methods are required to conduct a quantitative meta-analysis determining the brain region of plasticity on acupuncture for stroke in order to provide comprehensive evidence. Because systematic reviews and meta-analyses are important tools for summarizing specific topics that inform evidence-based practice in healthcare, guidelines, and policies in a comprehensive, meaningful manner [95]. Another drawback is the absence of SA as a control group, making it difficult to draw reliable conclusions and confirm acupuncture specificity. Third, the limited number of articles and small sample sizes limit the stability and reliability of this review; a large sample size and additional research are required. In addition, we include only peer-reviewed studies conducted in English, which could introduce some selection bias. Finally, since many studies did not perform correlation analyses between brain imaging and behavioral characteristics before and after treatment, it is difficult to clarify that changes in brain imaging are objective evidence of improvement in symptoms. Accordingly, future researchers should pay much attention to this research area. Therefore, to overcome the previously mentioned limitations, extensive research efforts need to be conducted in the future.

### 5.5. Conclusions

In summary, the cumulative evidences demonstrated that acupuncture could modulate neural plasticity of stroke, activating not only motor-related brain but also language-related and cognitive-related brain regions. Consequently, acupuncture therapy can enhance clinical recovery following a stroke. However, additional research is necessary to validate the results due to the scarcity of data.

## Figures and Tables

**Table 1:** Characteristics of the 24 included stroke patients studies.

*N*	Author (year)	Journal	Stroke information				MRI information	Acupuncture information			Data analysis	Experimental design
			Subjects	Affected side	Type of stroke, lesions (*N*)	Interval since stroke	Scanner	Intervention	Comparison	Acupoints		
1	Li et al. 2006 [39]	Journal of magnetic resonance imaging	12 stroke	The left side somatosensory deficits	IS, right hemispheric striatocapsular infarction	More than 6 months	1.5 T	MA	Stroke vs. HC	LI4 and LI11	SPM	Block/R(45 s)-S(45 s), 3 times
2	Schaechter et al. 2007 [34]	The journal of alternative and complementary medicine	7 stroke	NA	IS (5), HS (2), 5 left, and 2 right	4.6 ± 3.2 years	3 T	MA, SA (Streitberger needle, noninvasive control)	4 VA vs. 3 SA	N	GLM	RS/twice weekly for 10 weeks.
3	Li and Yang 2010 [40]	Complementary therapies in medicine	7 aphasia stroke/14 HC	The right side of the body	IS (6), HS (1); the occlusion of the middle cerebral artery, left hemisphere	More than 6 months	1.5 T	EA	Stroke vs. HC	SJ8	SPM	Block/R(45 s)-S(45 s), 3 times
4	Huang et al. 2011 [41]	NRR	12 stroke	The left hemiataxia and sensory disturbance	IS, right hemisphere	6.08 ± 6.40 months	3 T	MA, SA (nonacupuncture points in close proximity to acupuncture points)	6 VA vs. 6 SA	SJ5	ReHo	Block/S(30 s)-R(30sn), 6 times
5	Shen et al. 2012 [37]	ECAM	20 stroke	Basal ganglia, and completely or partially covered the internal capsules	IS	10.70 ± 11.13 hours	1.5 T	MA	10 acupuncture plus conventional treatments vs. 10 only conventional treatments	Du23, Du 20, EX-HN3, PC 6, and Sp 6	FA and ADC	RS/30 min, once a day, for 2 weeks
6	Cho et al. 2013 [42]	Chinese journal of integrative medicine	11 stroke/10 HC	The left side of the body	IS, right hemisphere	2-6 months	3 T	MA	Stroke vs. HC	LI11 and ST36	SPM	Block/R(30 s)-S(30 s), 3 times
7	Huang et al. 2013 [43]	Acupuncture in medicine	10 stroke	The right hemiplegia	IS, left hemispheric	1-12 months	3 T	MA	Before vs. after	SJ5	GLM	Tactile control (6 min)-R(5 min)-block/S(30 s)-R(30 s), 6 times
8	Chen et al. 2013 [44]	NRR	10 stroke/6 HC	The right hemiataxia	IS, left basal ganglia	5.30 ± 3.71 months	3 T	MA	Stroke vs. HC	SJ5	FC	Block/R(30s)-S(30s), 6 times
9	Bai et al. 2014 [31]	ECAM	9 stroke/8 HC	The left side of the body	IS, right hemispheric striatocapsular	2-12 weeks	3 T	MA	Before vs. after	GB34	FC	NRER/R(1 min)-S(1 min)-R(8 min)
10	Chen et al. 2014 [45]	PLoS one	24 stroke	The right hemiplegia	IS, left basal ganglia	1 month-1 year	3 T	MA, SA (tactile control, a noninvasive control)	12 VA vs. 12 SA	SJ5	FC	Block/rested for 5 min-SA(6 min 30 s)-R(6 min 2 s)-VA(6 min 30 s).
11	Qi et al. 2014 [46]	NRR	16 stroke	The right hemiataxia	IS, primarily in the left hemisphere	4.63 ± 3.85 months/4.63 ± 4.41 months	3 T	MA, SA (nonacupuncture points in close proximity to acupuncture points)	8 VA vs. 8 SA	SJ5	SPM	Block/R(30 s)-S(30 s), total 6 min 6 s
12	Xie et al. 2014 [32]	ECAM	9 stroke/8 HC	The left side of the body	IS, unilateral right-sided striatocapsular lesions	53.6 ± 41.6 days	3 T	MA	Stroke vs. HC	GB34	GLM and GCA	NRER/R(1 min)-S(1 min)-R(8 min)
13	Zhang et al. 2014 [47]	ECAM	8 stroke/10 HC	The left side of the body	IS, right hemispheric corona radiate, internal capsule, or basal ganglia infarction	2–12 weeks	3 T	MA	Stroke vs. HC	GB34	SPM	NRER/R(1 min)-S(1 min)-R(8 min)
14	Li et al. 2015 [48]	NRR	12 stroke	The right hemiataxia	IS, left basal ganglia	1 month-12 months	3 T	MA	Before vs. after	SJ5	SPM	Block/(30 s)-S(30 s), 6 times
15	Gao et al. 2015 [49]	Experimental and therapeutic medicine	10 stroke/10 HC	NA	IS, right subcortical	At least 6 months	3 T	MA	Stroke vs. HC	ST36	GLM	Block/(30 s)-S(30 s), 6 times
16	Chang et al. 2017 [50]	Wiener klinische Wochenschrift	43 poststroke motor aphasia	NA	Cerebral hemorrhage or cerebral infarction	14 days to 2 years	3 T	EA	22 EA vs. 21 WT	HT5, GB39	GLM	Block/R(30 s)-S(30 s), total 6 min 6 s
17	Fu et al. 2017 [51]	Medicine	19 stroke/17 HC	The left hemiplegia	IS, internal capsule, and neighboring regions in the right hemisphere	2 weeks to 6 months	3 T	MA	Before vs. after	GB34	ICA	RS/R(8 min 10 s)-needing in (1 min)-S(1 min)-R(8 min 10 s)
18	Ning et al. 2017 [52]	Frontiers in human neuroscience	18 stroke/20 HC	The left motor hemiparesis	First-ever IS, right subcortical stroke	Within 6 months after the onset	3 T	MA	Before vs. after	GB34	GLM, FC	NRER/R(1 min)-S(1 min)-R(8 min)
19	Li et al. 2017 [35]	Neural plasticity	17 stroke/14 HC	The right side of the body	IS, left basal ganglia, caudate nucleus, centrum semiovale, and lenticular nucleus	At least three weeks	3 T	MA	8 MA + drug vs. 9 drug	DU20, GB20, bilateral GB-39, LI-11, LI-4, ST-36, SP-6	FC	RS/two hours a day for 5 days a week, one week a course, continuous four courses
20	Wu et al. 2017 [38]	Journal of traditional Chinese medicine	21 stroke	NA	IS	Less than six months	3 T	MA	11 MA plus CT vs. 10 CT	DU20, GB20, LI11, LI4, GB34, ST36, SP6, and GB39	ReHo	RS/30 min, 2 times/week for 5 weeks
21	Wu et al. 2018 [36]	ECAM	21 stroke	NA	IS	Less than six months	3 T	MA	11 MA plus CT vs. 10 CT	DU20, GB20, LI11, LI4, GB34, ST36, SP6, and GB39	VBM	RS/30 min, 2 times/week for 5 weeks
22	Han et al. 2019 [33]	ECAM	22 stroke/22 HC	The left side of the body	IS, right-hemispheric subcortical infarct	41.68 ± 25.02 days	3 T	MA	Stroke vs. HC	GB34	FC	NRER/R(8 min 10 sec)-S(1 min)-R(8 min 10 sec)
23	Chen et al. 2020 [53]	Chinese journal of integrative medicine	10 stroke	The left side of the body	IS, the vascular occlusion in the right basal ganglia	1 month-3 years	3 T	MA	Before vs. after	LI11 and ST36	ReHo	RS/R(5 min)-S(15 min)-R(5 min)
24	Han et al. 2020 [54]	Neural plasticity	26 stroke/21HC	The left side of the body	IS, right hemispheric subcortical infarct	41.04 ± 29.71 days	3 T	MA	Stroke vs. HC	GB34	Graph theoretical network	RS/R(8 min 10 s)-S(60 s)-R(8 min 10 s)

Note: ADC: apparent diffusion coefficient; BOLD: blood oxygen level-dependent; CT: conventional treatments; EA: electroacupuncture; ECAM: Evidence-Based Complementary and Alternative Medicine; FA: fractional anisotropy; FC: functional connectivity; GCA: granger causality analysis; GLM: general liner model; HC: health controls; HS: hemorrhagic stroke; IS: ischemic stroke; MA: manual acupuncture; *N*: number; NA: not applicable; NRER: nonrepeated event-related; RS: resting state; NRR: Neural Regeneration Research; ReHo: regional homogeneity; TBSS: tract-based spatial statistics; R: rest; s: seconds; SA: sham acupuncture; S: stimulation; VA: verum acupuncture; min: minutes; VBM: voxel-based morphometry; WT: waiting list; ICA: independent component analysis; Y: yes. A.

**Table 2:** Characteristics of the five included animal studies.

Study (years)	Journal	Stroke information			MRI information	Groups	Acupuncture information		Data analysis
		Main symptoms	Species	The affected side	Magnet strength (T)		Treatment group	Acupoints	
Zhang et al. 2011 [55]	Brain injury	Middle cerebral artery occlusion (MCAO) model	SD rats, after 24 hours of the surgery	Left side	NA	(1) MCAO, *n* = 6 (2) MCAO + EA, *n* = 6	EA, 30 minutes	DU20	DWI
Wu et al. 2012 [56]	Acupuncture in medicine	Transient middle cerebral artery occlusion (tMCAO)	SD rats, after 30 minutes of the surgery	Left side	1.5-T	(1) SC, *n* = 12 (2) tMCAO, *n* = 12 (3) tMCAO + EA, *n* = 12	MA, 30 minutes for 28 days	DU20, DU14, LI10, and ST36	ADC value and the FA
Liang et al. 2017 [57]	Journal of stroke and cerebrovascular diseases	Motor impairments/middle cerebral artery occlusion (MCAO)	SD rats, after 24 hours of the surgery	Left side	7.0 T	(1) SC, *n* = 12 (2) MCAO, *n* = 9 (3)MCAO + EA, *n* = 9	EA, 30 minutes per day for 7 consecutive days	ST36 and LI11	ReHo
Wen et al. 2018 [58]	Journal of stroke and cerebrovascular diseases	Middle cerebral artery occlusion induced cognitive deficit (MICD)	SD rats, after 24 hours of the surgery	Left side	7.0 T	(1) SC, *n* = 12 (2) MICD, *n* = 12 (3) MICD + EA, *n* = 12	EA, 30 minutes per day for 14 consecutive days	DU20 and DU24	ALFF
Li et al. 2021 [81]	Acupuncture in medicine	Motor impairments/middle cerebral artery occlusion (MCAO)	SD rats, after 24 hours of the surgery	Left side	7.0 T	(1) SC, *n* = 12 (2) MCAO, *n* = 9 (3) MCAO + EA, *n* = 9	EA, 30 minutes per day for 14 consecutive days	LI11 and ST36	FC, left motor cortex as the seed region

Note: ALFF: amplitude of low-frequency fluctuations; DWI: diffusion-weighted imaging; SC: sham-operated control; SD: Sprague-Dawley rats.

## Data Availability

Our data are from the published literature.
